# Spatio-temporal prevalence of malaria and anaemia in relation to agro-ecosystems in Mvomero district, Tanzania

**DOI:** 10.1186/s12936-019-2859-y

**Published:** 2019-07-09

**Authors:** Susan F. Rumisha, Elizabeth H. Shayo, Leonard E. G. Mboera

**Affiliations:** 1 National Institute for Medical Research, 3 Barack Obama Drive, P.O. Box 9653, Dar es Salaam, Tanzania; 2 SACIDS Foundation for One Health, Sokoine University of Agriculture, Chuo Kikuu, P.O. Box 3297, Morogoro, Tanzania

**Keywords:** Malaria, Parasitaemia, Anaemia, Agro-ecosystems, Tanzania

## Abstract

**Background:**

Agro-ecological systems have been associated with increased malaria intensity. This study determined association between different agro-ecological systems, prevalence of malaria parasitaemia and anaemia in Mvomero district, Tanzania.

**Methods:**

The study was carried out in three agro-ecosystems namely, savannah, rice-irrigation, and sugarcane. Malaria and anaemia prevalence were measured in four seasons of a year. Villages were categorized according to environmental characteristics, proportion of water-shaded areas and agro-ecosystems. Mixed-effects logistic regression analysis was used to determine factors associated with malaria infection.

**Results:**

A total of 7888 individuals were involved with the overall malaria prevalence of 34.4%. *Plasmodium falciparum* was the dominant (99.52%) malaria species. Malaria prevalence was highest (42.9%) in children of 10–15 years of age, and significantly low during dry and hot season. Of the infected individuals, 78.1% were from rice-irrigation, 18.7% savannah and 3.2% sugarcane ecosystem. Individuals living in villages with high levels of water-shaded areas had highest malaria risk. Over three-quarters (78.9%) of the individuals slept under a mosquito net, with the highest (88.5%) coverage among individuals in sugarcane ecosystem. On average 47.1% of the children were anaemic. Anaemia was more prevalent (60.5%) among individuals in the savannah than in the rice-irrigation (48.2%) or sugarcane communities (23%). Analysis indicated that ecosystems and levels of water-shaded area were highly correlated, and altered levels of malaria infection. Gender, age, mosquito net-use, and season were other significant determinants of *P. falciparum* infection. Males had higher odds than females (OR = 1.16, 95% CI 1.05, 1.29). The risk for children 6–9 years and older children (10–15 years) was over 50% and 24%, respectively, higher compared to young ones (0–5 years). Use of mosquito net reduced malaria risk by 26%. The risk of infection was higher during dry and cool season (OR = 1.92, 95 %CI 1.66, 2.23) compared to other seasons. Living in villages with high level of water-shaded areas increased the chances of getting malaria up to 15 times than living in drier areas. Similarly, infection odds increased when living in savannah and rice-irrigation ecosystems than in the sugarcane ecosystem.

**Conclusions:**

Findings show significant variations in malaria prevalence between communities living in different agro-ecosystems within the same district. Local malaria control strategies should consider these variations and liaise with agricultural experts while designing interventions to maximize effectiveness.

## Background

Malaria remains a major public health problem in the tropic and sub-tropic regions, with approximately 216 million cases and half a million deaths reported annually [1, 2]. About 90% of the malaria cases and deaths occur in Africa [1, 3–6]. Tanzania stands among the top ten countries with high malaria transmission and population at risk in eastern and southern African region [1, 7, 8]. The disease is endemic in almost all parts of the country, with over 95% of the population at risk of infection. In Tanzania, malaria contributes to 26% of all outpatient attendances, resulting in an estimated 7.7 million confirmed and clinical malaria cases annually [7, 9]. The disease accounts for 33.4–42.1% of all hospital admissions and it is the leading cause of hospital deaths in Tanzania [1, 10]. Malaria has also been confirmed to attribute to anaemia in children [11–13].

The prevalence and incidence of malaria vary from place to place and times of the year. Both climatic and non-climatic factors affect malaria transmission. Climatic factors, including temperature, rainfall and relative humidity, greatly influence the pattern and levels of malaria [14–18]. Non-climatic factors that influence malaria risk include types of vector, species of malaria parasite, host immunity, insecticide and drug resistance, environmental development and urbanization, population movements, and other socio-economic factors including livelihoods [19–30]. In sub-Saharan Africa, agricultural practices and other livelihoods activities have been described to have impact on malaria transmission [15–23], either through increasing mosquito reproduction and survival or exposure of humans to mosquito bites [22, 31]. To maximize productivity, there had been intensification of food production methods, increased crop irrigation and forest-clearing, all of these contributed to an increase in mosquito breeding habitats [32–34]. For instance, swampy rice cultivation methods and use of flooded paddies create favourable breeding sites for *Anopheles gambiae* which is the main malaria vector [35, 36], while agricultural activities in savannah areas favour *Anopheles funestus* [37]. Moreover, there are evidence that large-scale development projects contribute to increasing in malaria transmission intensities. These include the introduction of new agricultural practices, infrastructural development programmes, such as water resource management, road constructions and maintenance, brick making and mining [38–41].

Like in most African countries, over two-third of the population in Tanzania lives in rural areas, where majority are involved in agricultural activities [9, 42]. Communities, depending on their locations and crops cultivated, opt for specific farming practices to optimize agricultural productivity [43, 44]. These options alter risk of acquiring malaria infection and transmission intensities. Although the density of malaria vectors has been reported to vary among areas within small proximity but practicing different farming practices [34, 36, 45], a study in Kenya has shown low malaria prevalence in areas where irrigation is practised than in non-irrigation areas [32]. These multidirectional findings result into complexity when deciding on disease control and vector management strategies [22, 32]. To complement and sustain the gains achieved in malaria control, it is crucial that the farming communities and those making decisions in health and agricultural sectors understand the impact of various agricultural practices and agro-ecosystems on malaria transmission dynamics [46–48]. However, there are limited number of studies that have established the micro effect of choices on ecosystems and farming practices on malaria burden [13, 24, 47, 49–51]. As a result, knowledge on their associations remain relatively weak. Such information is critical for evidence-based actions, to guide health policy and planning, such as promotion of intersectoral strategies in malaria control and developing area-specific community-tailored control strategies [52, 53]. This study was carried out to determine the relationship between different agro-ecological systems and the prevalence of malaria and anaemia among rural communities in Mvomero district in east-central Tanzania.

## Methods

### Study area

This study was carried out in Mvomero district in east-central Tanzania. The district is located between latitudes 5–8°S and longitudes 37–39°E, and lies on the foothills of Nguru Mountains to the north-west and Uluguru Mountains to the south-east. The area lies on the Wami River basin. Most of the inhabitants (80%) earns their livelihoods from agriculture. The main economic activities include rice farming, sugarcane plantations and livestock production. Administratively the district is divided into four divisions, 30 wards and 101 villages. Total human population is estimated at 312, 109 with an average growth rate of 2.6% [54]. Rainfall is high (1146 mm per annum) and bimodal with a relatively short dry spell between June and September. The mean maximum temperature is 31 °C (October to March); whereas the mean minimum temperature is 19 °C (June to September).

**Fig. 1:**
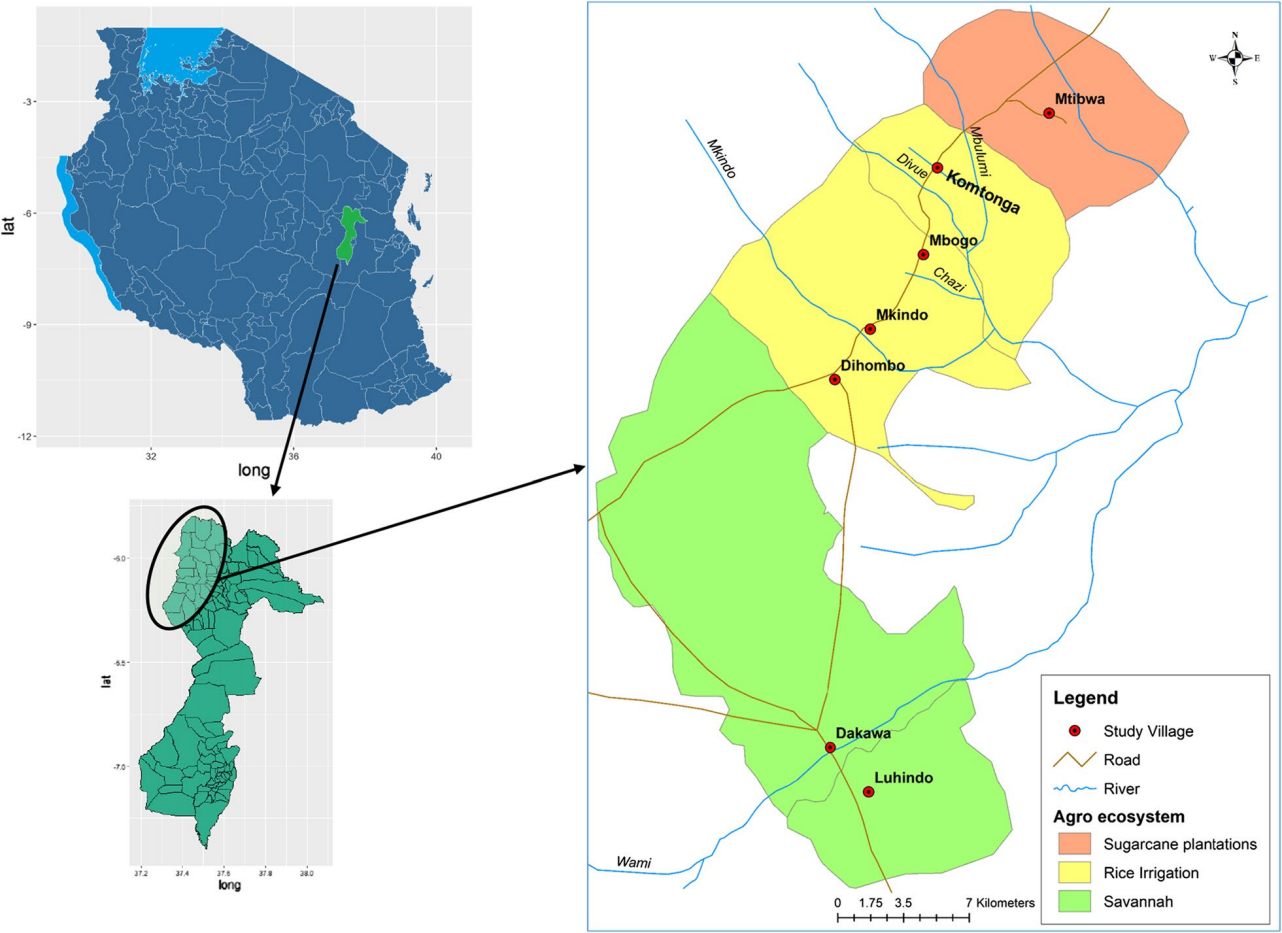
Location of study villages with their agro-ecosystems in Mvomero district, Tanzania

Two divisions, namely Mvomero and Turiani, lying at 293–379 m above sea level were selected for the study. Within the division, seven villages (Luhindo, Dakawa, Dihombo, Mkindo, Mbogo, Komtonga and Mtibwa) characterized by three agro-ecosystems, savannah, rice-irrigation and sugarcane plantation were included in the study (Fig. 1). The agro-ecosystems were grouped based on vegetation cover and main agricultural practices performed by the community members in the respective villages. The total area covered by the study was approximately 20 km × 60 km. Other important water bodies existing within these villages include four rivers i.e. Wami, Mkindo, Mbulumi and Divue. These rivers are the main sources of water for irrigation (Fig. 1).

### Study design

**Fig. 2:**
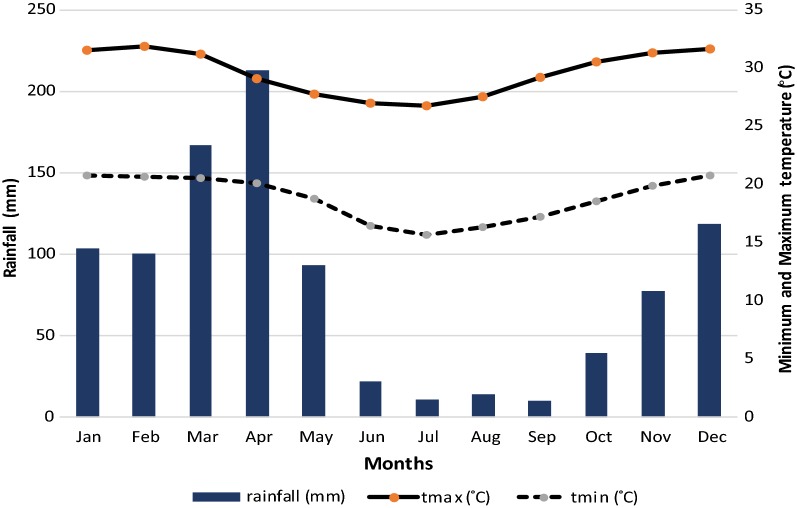
Pattern of rainfall and temperature in Mvomero district (Source: http://maproom.meteo.go.tz (Temperature data average for 1961–2014; rainfall average for 1983–2010; tmax = maximum temperature; tmin = minimum temperature)

This was a cross-sectional survey conducted in 4 phases during four climatic seasons of August (dry and cool season) 2004, November 2004 (short rains), February 2005 (dry and hot season) and May 2005 (long rains) (Fig. 2). With assistance from village authorities, a specified number of households was selected randomly and the selected families were invited to participate in the study. The sampling frame was the updated household listing provided by the village leadership. Primary schools within selected villages were visited for convenience inclusion of school-children in the study; each village had only one primary school. The minimum number of individuals required for the survey was estimated using a formula for calculating sample size for cross sectional prevalence studies [55, 56]. A conservative malaria prevalence of 50% was considered [57] as precise estimates for the prevalence for Mvomero district was not available at times when the study was designed. With a margin error of 5% and 95% confidence level (α = 5%, Z = 1.96), and a design effect of 1.5 to account for variation on prevalence between sites, a minimum sample size of 576 households was established to be sufficient for the study. A probability proportional to size (total number of households within a village) was used to obtain sample size for each village. On average, 2 to 4 participants (average of 3) were expected from each household, hence expected individuals to be included in each survey phase were estimated at 1728. A nonresponse rate of 10% was considered to give a minimum sample of 1900 per round of the survey. Community malaria prevalence and related parameters have been estimated using school children [41, 58–62] and it is convenient to study this group in terms of costs and logistics; with that, the sample size was split between community and schools into a ratio of 30%:70% for implementation. That was also supported by the fact that, if screening was to be done simultaneously at both sites at the same day, most children aged 6–15 years will be at school hence targeting schools as point of collection was convenient.

### Environment assessment and categorization of study sites

Environment assessment survey was conducted in all seven villages. Departments of Land, Natural Resources and Agriculture were consulted and discussions were held. Physical visits and observations were made to the study villages. Information documented include land use and land cover patterns, i.e. farming systems (crop/livestock production), infrastructure, natural resources, water sources and reservoir, ecosystems, water-shaded, type of houses, environmental cleanliness and weather patterns. Water-shaded area was defined as those reported to hold standing water continuously for 4 to 6 months a year. It is a proxy for surface water availability at micro-level—which is associated with providing conducive mosquito breeding sites. Results of the environmental assessment were used to stratify the study area into three agro-ecosystems; (i) savannah, including the villages of Luhindo and Dakawa; (ii) rice-irrigation which comprised Dihombo, Mkindo, Komtonga and Mbogo; and (iii) sugarcane plantations in Mtibwa village. Within the rice-irrigation ecosystem, flooding irrigation, which is a traditional method, was common practiced in Komtonga Dihombo and Mbogo. In this area, farmers use temporary made water canals to irrigate their rice fields, these are constructed each season based on how the paddies are needed. Mkindo village adopted a formal irrigation scheme system using well-constructed canals. There is a main canal fed from the river (set by gravity) and local farmers are connected to irrigate their farms. The communities in savannah agro-ecosystem cultivates rice and maize, in addition, they are involved in livestock keeping. Dakawa village is characterised by large-scale rice farms with well-maintained water scheme. The main season for rice cultivation starts from late November to February (preparations and planting) to May–July (harvesting) and the off-season runs from June through December. Most farmers cultivate during the main season with a few doing both seasons [13, 33]. The village authorities identified areas for cultivations where community members either buy and own the land or hire from the authorities on annual basis. The average farm size varies with the type of cropping system, hence varies from village to village and range from 2 to 3 ha. Farmers reported to walk a range of 1–3 h to their farms. Information on amount of permanent water bodies observed, type of vegetation and farming systems was used to categorize the villages into levels of water shaded areas. The list of villages in a decreasing order of water-shaded area is Komtonga, Mkindo, Mbogo (*high level*); Dihombo, Luhindo, Dakawa (*medium to low level*) and Mtibwa (*dry land*). The study area characteristics have been extensively described elsewhere [34, 51, 63].

### Malariometric surveys

Clinical examination and screening for malaria parasites for community members was done at a central point in respective villages. Primary schoolchildren were examined at their own schools under the supervision of school head teachers. In each school, approximately 200 (average per schools taken to reach the needed sample) children were examined for malaria parasites and about 15% examined for haemoglobin levels. To get age representation, children were included from all seven classes with the needed sample equally split and balancing for gender groups. A systematic sampling procedure was applied to a class register (separately for boys and girls) to select children. All children below 15 years both in and out of school were examined for splenomegaly through the palpation of the spleen in a horizontal position by the research physician [64].

History of fever and malaria symptoms were recorded and an axillary temperature was measured with a digital thermometer. Thick and thin blood smears were collected and stained with Giemsa and examined with a binocular microscope with an oil immersion lens to quantify the parasitaemia. Parasitaemia was measured counting the number of asexual parasites against the number of leukocytes in the blood film, based on a putative count of 8000 leukocytes per microlitre. The number of asexual parasites was counted against 200 leukocytes using a hand tally counter. A slide was considered negative if no parasite were seen after scanning 200 fields. Children with positive test results were followed and treated following recommended treatment guidelines and referred to the nearest health facility when necessary. Each individual was asked on whether he/she slept under a mosquito net a night before the survey. Parents or caretakers were consulted to confirm information on use of mosquito net provided by very young children (between 3 and 5 years). Among the schoolchildren, pictures of mosquito nets (with different colours) were used to illustrate for those who had difficulties in understanding the question on net use.

### Data analysis

The data were double-entered, cleaned and validated in Epi-Info 6.04 (CDC Atlanta, USA, 2001). Descriptive and statistical analysis were carried out in MS Excel and Stata 13 (StataCorp. 2013), respectively. Characteristics of the study participants were examined and presented. Mean with standard deviations and median values (with interquartile ranges, IQR) were calculated for continuous variables (where appropriate) while proportions were used for categorical variables. A binary variable describing malaria status was created to take value of 1 if malaria was present and 0 otherwise. Malaria prevalence levels were compared between different characteristics. An individual was considered to have fever if the body temperature was ≥ 37.5 °C. Normal haemoglobin level was taken when concentration was ≥ 11 g/dl while a person with value less than 11 g/dl was considered anaemic. Mild anaemia was defined if haemoglobin was ≥ 10–≤ 11 g/dl, moderate between ≥ 7 and < 10 g/dl and severe anaemia if haemoglobin was less than 7 g/dl [7]. Two-sample test of proportions, $\chi^{2}$-test, Fisher’s exact and *t* test were used for comparison where appropriate. Cuzick’s test of trend [65] was used to determine ordered effect of levels water-shaded area on malaria prevalence. The statistical level of significance was set at p < 0.05.

A mixed-effects logistic regression model was used to determine association between malaria infection status and other factors including climate seasons, agro-ecosystems, levels of water-shaded area, use of mosquito net and demographic characteristics. The model includes village-specific random effects to account for clustering. Pearson’s correlation coefficient was used to detect highly correlated covariates (r > 0.8). To select which covariates to retain between a correlated pair, the two were ranked based on their goodness of fit assessed through Akaike information criteria (AIC) [66] and pseudo-R^2^. The best fitting one was used in the final model. The smaller the AIC and the higher the pseudo-R^2^ indicated the better the variable. The Variance Inflation Factor (VIF) and Farrar-Glauber test (F-G test) for multicollinearity [67] were performed and variable with VIF > 4 were omitted in the model. Odds ratios (with 95% confidence intervals) of all variables used in the final model are presented to indicate the strength and direction of the association.

### Ethical considerations

Ethical clearance was sought from the Medical Research Coordinating Committee of the National Institute for Medical Research, in Tanzania (NIMR/HQ/R.8a/Vol IX/297). Permission to conduct the study was sought from Mvomero district authority. Oral informed consent was obtained from the community members ≥ 18 years and assent was obtained from parents and teachers in the respective households or schools.

## Results

### Malaria parasitaemia and splenomegaly

A total of 7888 individuals were involved in the four cross-sectional malariometric surveys. Among these 52.1% were females, most (45.1%) aged between 6 and 9 years and over three quarters (78.9%) of respondents reported use of mosquito net during the previous night (Table 1).

**Table 1:** Characteristics of study individuals by malaria infection status (N = 7888)

Variable	Negative N (%)	Positive N (%)	Total N (%)	p-value
Gender^a^				
Female	2781 (53.8)	1327 (48.9)	4108 (52.1)	< 0.001
Male	2389 (46.2)	1389 (51.1)	3778 (47.9)	
Age category (years)				
0–5	508 (9.8)	246 (9.1)	754 (9.6)	< 0.001
6–9	2300 (44.5)	1258 (46.3)	3558 (45.1)	
10–15	1377 (26.6)	1037 (38.2)	2414 (30.6)	
> 15	987 (19.1)	175 (6.4)	1162 (14.7)	
Study subjects				
Community members	1723 (33.3)	492 (18.1)	2215 (28.1)	< 0.001
Schoolchildren	3449 (66.7)	2224 (81.9)	5673 (71.9)	
Usage of mosquito net				
No	1005 (19.4)	663 (24.4)	1668 (21.2)	< 0.001
Yes	4167 (80.6)	2053 (75.6)	6220 (78.9)	

^a^2 individuals missing information on gender

Overall malaria prevalence was 34.4% (2716/7888). It was similar among female and male individuals, apparently, the infection was concentrated in schoolchildren (p-value < 0.001, Two-sample test of proportions). Children aged 6–9 years carried the most burden followed by those aged 10–15 years. Malaria infection rate was higher among individuals not using nets than those reported to use (39.7% vs. 33.0%, p-value < 0.001). School-children < 5 years reported higher net usage as compared to those of the same age from the community group (89.2% vs. 82.3%, p-value = 0.025). In contrary, individuals 10–19 years sampled from the community reported higher usage than those in sampled at their schools (85.1% vs. 73.8%, p-value < 0.001). Malaria parasite prevalence was highest (42.9%) in the age group 10–15 years and lowest among > 15 years old (15.1%) (< 0.001). The prevalence levels in other age groups was 32.6% and 35.4% in ≤ 5 years and 6–9 years old, respectively. Prevalence within rounds were 36.7% (767/2088) round 1, 38.2% (730/1913) round 2, 26.6% (508/1913) round 3 and 36.0% (711/1974) during round 4.

Proportionally, less infected individuals were observed during round 3 (February 2005, dry and hot season) as compared to other periods (p-value < 0.0001). Of the 2716 individuals with malaria parasites, 507 (18.7%) were from savannah (Luhindo, Dakawa), 2122 (78.1%) rice-irrigation (Dihombo, Mkindo, Komtonga and Mbogo) and 87 (3.2%) sugarcane ecosystem (Mtibwa). Significant differences (p-value < 0.0001) in malaria prevalence were observed between the three agro-ecosystems with individuals from the sugarcane ecosystem having the lowest rate (Table 2).

**Table 2:** Distribution of malaria infection by study round (R), village, ecosystem and watershed

Variable	Negative N (%)	Positive N (%)	Total N (%)	p-value^a^
Survey rounds (R)				
R1 (August: dry and cool)	1321 (25.5)	767 (28.2)	2088 (26.5)	< 0.001
R2 (November: short rains)	1183 (22.9)	730 (26.9)	1913 (24.3)	
R3 (February: dry and hot)	1405 (27.2)	508 (18.7)	1913 (24.3)	
R4 (May: long rains)	1263 (24.4)	711 (26.2)	1974 (25.0)	
Village				
Komtonga	380 (7.4)	746 (27.5)	1126 (14.3)	< 0.001
Mkindo	672 (13)	470 (17.3)	1142 (14.5)	
Mbogo	655 (12.7)	557 (20.5)	1212 (15.4)	
Dihombo	789 (15.3)	206 (7.6)	995 (12.6)	
Luhindo	776 (15)	349 (12.9)	1125 (14.3)	
Dakawa	786 (15.2)	301 (11.1)	1087 (13.8)	
Mtibwa	1114 (21.5)	87 (3.2)	1201 (15.2)	
Ecosystem				
Savannah	1575 (30.5)	507 (18.7)	2082 (26.4)	< 0.001
Rice-irrigation	2483 (48)	2122 (78.1)	4605 (58.4)	
Sugarcane	1114 (21.5)	87 (3.2)	1201 (15.2)	
Water shed area				
High	1707 (33)	1773 (65.3)	3480 (44.1)	<0.001
Medium–Low	2351 (45.5)	856 (31.5)	3207 (40.7)	
Dry	1114 (21.5)	87 (3.2)	1201 (15.2)	
Total	5172 (65.6)	2716 (34.4)	7888	

^a^p-values estimated from Chi square test of association

**Fig. 3:**
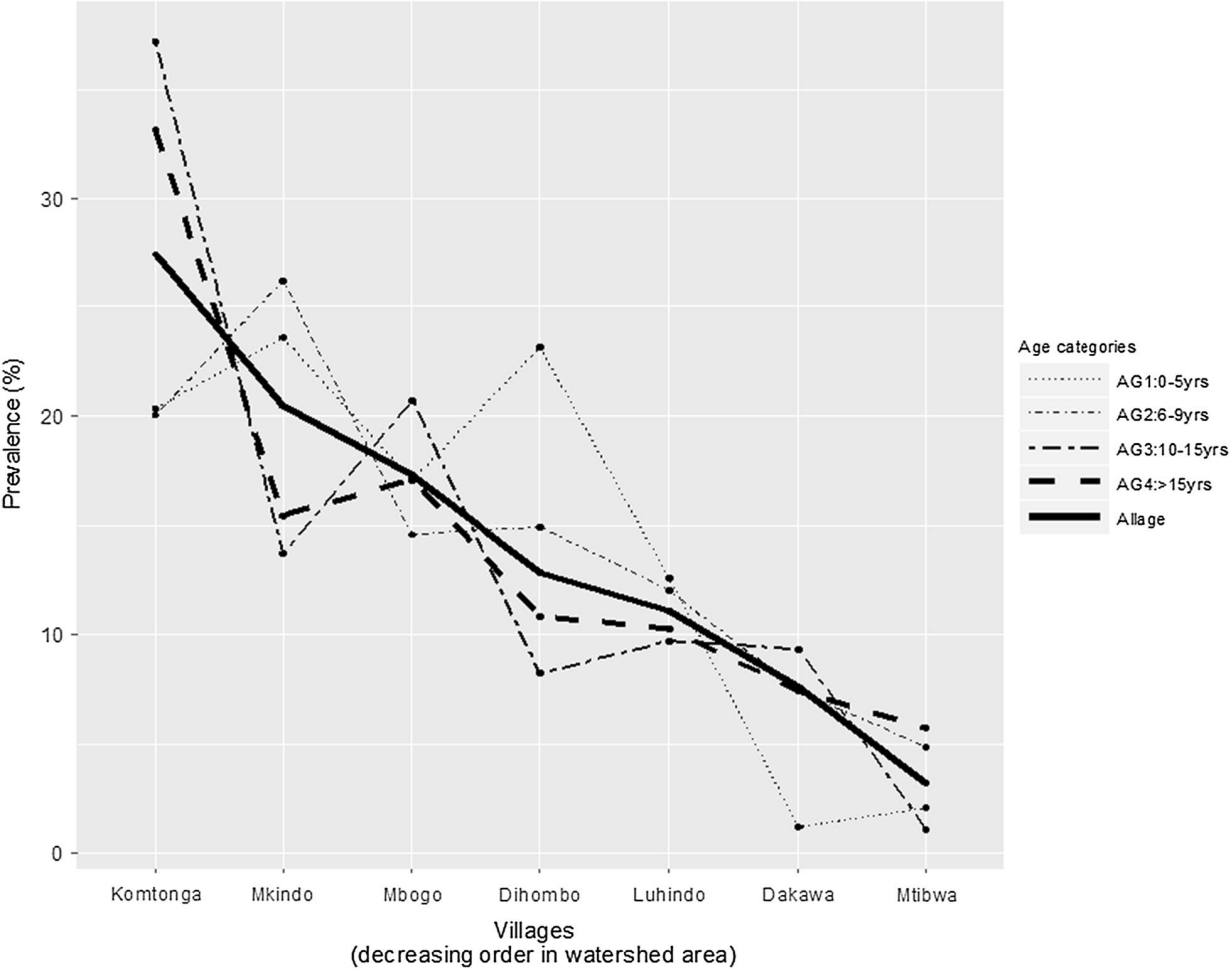
Trends of malaria infection rate by village ordered in a decreasing watershed area

The highly water shaded areas indicated the highest prevalence. Trend across villages was positive and highly significant, with individuals in villages with high levels of water-shaded areas (Komtonga and Mkindo) having highest malaria risk (Cuzick’s test, p-value < 0.001, Table 3, Fig. 3). The patterns by different age categories show a very similar trend.

**Table 3:** Number of individuals screened, mean age, *Plasmodium falciparum* (*Pf*) gametocyte rate, parasite prevalence, spleen rate and geometric mean parasite density (GMPD) by agro-ecosystems and village

Ecosystem	Village	No. screened	Mean age	*P. falciparum* N (%)	GMPD/μl	Gametocyte rate	Spleen rate^a^ N (%)
Savannah	Luhindo	1087	12.01	301 (27.7)	228.48	1.20	288 (27.1)
	Dakawa	995	13.50	206 (20.7)	161.44	0.98	105 (10.6)
	Total	2082		507 (24.4)			393 (19.1)
Sugarcane	Mtibwa	1201	11.71	87 (7.2)	434.25	2.17	58 (4.8)
Rice-irrigation	Mkindo	1212	12.09	557 (46.0)	204.46	4.60	199 (16.4)
	Dihombo	1125	13.42	349 (31.0)	278.64	1.73	198 (17.6)
	Mbogo	1142	12.78	470 (41.2)	322.13	1.29	204 (17.9)
	Komtonga	1126	14.18	746 (66.3)	257.24	1.34	330 (29.5)
	Total	4605		2122 (46.1)			931 (20.3)
Overall		7888	12.79	2716 (34.4)	269.52	1.78	1382 (17.6)

^a^Done for children children ≤ 15 years

The median body temperatures of the examined individuals was 36.9 °C (Interquartile range, 36.7–37.2 °C). Of the individuals whose body temperature measure was taken (n = 7856), 396 (5.0%) had fever (axillary temperature ≥ 37.5 °C). The largest proportion of those with fever was observed during the dry and cool season of August 2004 (n = 130, 32.8%) and dry and hot season of February 2005 (n = 117, 2935%) (p < 0.001). The proportion of individuals with fever (≥ 37.5 °C) was significant less in the community from savannah agro-ecosystem (Savannah = 5.6%; Rice-irrigation = 7.4%; sugarcane = 8.0%, p-value = 0.0719), and, a significant proportion (61%) was from community living in rice-irrigation ecosystems. Fever was more profound in individuals with malaria (8.3%) as compared to those who were not infected (6.1%), p-value = 0.002.

Enlargement of the spleen among children ≤ 15 years was observed mostly among individuals living in rice-irrigation agro-ecosystem than in the other ecosystems (Table 3). When spleen rate was analysed by age groups, it was observed to vary from 17.4%, 19.9%, 20.0% and 5.6% for ≤ 5 years, 5–9 years, 10–15 years and > 15 years old, respectively. Spleen enlargement was significantly higher in malaria infected individuals (30.4%, 95% CI 28.6–32.2%) than non-infected (14.3%, 95% CI 13.3–15.4%).

The average proportion of gametocytaemic individuals (proportion of those with asexual parasites who also had asexual-stage gametocytes) was 1.8%. It was higher in Mkindo (4.6%) and lower in Dakawa (0.98%). It was 1.7%, 2.2%, 1.3%, 1.2% and 1.3% in Dihombo, Mtibwa, Komtonga, Luhindo and Mbogo, respectively (Table 3).

*Plasmodium falciparum* was the dominant parasite species (99.5%). Mixed infections accounted for 0.5% (13/2716) of the total malaria parasite infections. Mixed infections due to *P. falciparum* + *Plasmodium malariae* were more common (0.4%) than due to *P. falciparum* + *Plasmodium ovale* (0.07%). Mixed *P. falciparum* + *P. malariae* infections were observed among persons living in four of the seven study villages. Mixed *P. falciparum* + *P. ovale* infections were only observed among individuals in Dihombo village. One individual from the sugarcane ecosystem was found to be infected with *P. malariae* (Table 4).

**Table 4:** Malaria parasite species composition by agro-ecosystem and village in Mvomero district

Ecosystem	Village	*Pf*	*Pf* + *Pm*	*Pf* + *Po*	*Pm*	Total
Savannah	Luhindo	301 (100%)	0	0	0	301
	Dakawa	205 (99.5%)	1 (0.5%)	0	0	206
Sugarcane	Mtibwa	82 (94.3%)	4 (4.6%)	0	1 (1.2%)	87
Rice-irrigation	Mkindo	557 (100%)	0	0	0	557
	Dihombo	347 (99.4%)	0	2 (0.6%)	0	349
	Mbogo	467 (99.4%)	3 (0.6%)	0	0	470
	Komtonga	744 (99.7%)	2 (0.3%)	0	0	746
	Total	2703 (99.5%)	10 (0.4%)	2 (0.07%)	1 (0.04%)	2716

Key: *Pf* = *P. falciparum*; *Pm* = *P. malariae*; *Po* = *P. ovale*

### Mosquito net utilization

On average, over three-quarters (78.9%; range = 69.7–88.5%) of the individuals slept under a mosquito net the previous night. The largest mosquito net coverage was in Mtibwa (88.5%) followed by Mbogo (85.3%), Dihombo (80.6%), Komtonga (80.3%), Mkindo (75.3%) and the lowest coverage was in Dakawa (69.7%). Of the total persons (6200/7893) reported using nets, 635 were < 5 years, 2831 6–9 years, 1818 10–15 years and 940 were ≥ 15 years of age. However, zooming within these age groups, < 5 years had the highest proportion (84.1%) of those sleeping under mosquito net. The proportions for the other age groups were 79.6%, 75.3% and 80.7% for 6–9, 10–15 and > 15 years old, respectively.

### Prevalence of anaemia among children

Overall mean haemoglobin (Hb) level was 10.8 g/dl (range = 3.6–15.2 g/dl). The mean Hb level was similar among female (10.83 ± 1.91 g/dl) and male (10.86 ± 1.81 g/dl) children. On average 47.1% of the children were anaemic. Anaemia levels were similar between males (45.8%) and females (47.9%). Age group most affected by anaemia was 10–15 years (49.3%). Anaemia was more prevalent (60.5%) among individuals in the savannah than in the rice-irrigation (48.2%) or sugarcane farming communities (23%) (Table 5). However, severe anaemia was more prevalent among children in rice-irrigation village of Mbogo. Anaemia level was significantly higher in malaria infected individuals (55.2%, 95% CI 50.2–60.1%) than non-infected (41.6%, 95% CI 37.8–45.4%). Significant differences in anaemia levels were detected between infected and non-infected individuals in rice-irrigation agro-ecosystem and specifically in Komtonga village.

**Table 5:** Mean haemoglobin (Hb) level (g/dl) and the prevalence (%) of anaemia among children by agro-ecosystems and villages

Ecosystem	Village	Mean Hb	Range Hb	Normal (≥ 11 g/dl)	Mild (11.0–10.9/dl)	Moderate (7.0–9.9/dl)	Severe (4.0–6.9/dl)	Total
Savannah	Luhindo	10.4	5.8–15.2	60 (40.0)	33 (22.0)	50 (33.3)	7 (4.7)	150
	Dakawa	10.4	5.5–13.6	58 (38.9)	40 (26.9)	44 (29.5)	7 (4.7)	149
	Total	10.4	5.5–15.2	118 (39.5)	73 (24.4)	94 (31.4)	14 (4.7)	299
Sugarcane	Mtibwa	11.7	4.8–15.2	154 (77.0)	31 (15.5)	12 (6.0)	3 (1.5)	200
	Total	11.7	4.8–15.2	154 (77.0)	31 (15.5)	12 (6.0)	3 (1.5)	200
Rice-irrigation	Mkindo	10.4	4.4–14.3	69 (45.4)	24 (15.8)	49 (32.2)	10 (6.6)	152
	Dihombo	11.1	4.9–14.9	92 (60.9)	31 (20.4)	24 (15. 9)	5 (3.3)	152
	Mbogo	10.5	3.6–14.2	47 (47.5)	24 (23.2)	19 (19.2)	10 (10.1)	100
	Komtonga	10.9	4.8–14.2	63 (52.1)	29 (24.0)	26 (21.3)	4 (3.3)	122
	Total	10.7	3.6–14.2	271 (51.8)	108 (20.3)	118 (22.4)	29 (5.5)	526
Overall	Total	10.8	3.6–15.2	543 (52.9)	212 (20.7)	224 (21.8)	46 (4.5)	1025

### Determinants of malaria

Mixed effects logistic regression models were fitted to determine factors associated with malaria infection in the study population. Agro-ecosystems, levels of water-shaded area and village variables were highly correlated. Based on the criteria set, the levels of water-shaded area was retained. The variable that distinguish the schoolchildren and general community members was omitted due to high variation initiation factor value.

Gender, age, use of mosquito nets, and season of the year were found as significant determinants for *P. falciparum* malaria infection (Table 6). Male individuals had 16% higher odds of having malaria than females (OR = 1.16, 95% CI 1.05, 1.29). Children 6–9 years had the risk of over 50% compared to the very young ones (0–5 years). The risk for the older children (10–15 years) was 24% higher. These results demonstrated high vulnerability to malaria infection among adolescents. Adults presented an age favour and indicated a less risk (by 68%) to get malaria compared to 0–5 years children (OR = 0.32, 95% CI 0.25, 0.40).

**Table 6:** Mixed-effects logistic regression model on determinants of malaria infection

Variable	Unadjusted		Adjusted	
	OR	(95% CI)	OR	(95% CI)
Gender				
Male	1.22	(1.11, 1.34)***	1.16	(1.05, 1.29)**
Female	1.00		1.00	
Age category (years)				
0–5	1.00		1.00	
6–9	1.13	(0.96, 1.33)	1.5	(1.25, 1.79)***
10–15	1.56	(1.31, 1.85)***	1.24	(1.03, 1.5)**
> 15	0.37	(0.29, 0.46)***	0.32	(0.25, 0.4)***
Usage of mosquito net				
No	1.00		1.00	
Yes	0.75	(0.67, 0.83)***	0.74	(0.66, 0.84)***
Subject group^a^				
Schoolchildren	2.26	(2.02, 2.53)***		–
General community	1.00		1.00	
Season (round of survey)				
Round 1 (August: dry and cool)	1.61	(1.4, 1.84)***	1.98	(1.71, 2.3)***
Round 2 (November: short rains)	1.71	(1.49, 1.96)***	1.92	(1.65, 2.24)***
Round 3 (February: dry and hot)	1.00		1.00	
Round 4 (May: long rains)	1.56	(1.36, 1.79)***	1.69	(1.45, 1.96)***
Village^a^				
Komtonga	25.14	(19.56, 32.3)***		–
Mkindo	10.89	(8.52, 13.92)***		–
Mbogo	8.96	(6.99, 11.48)***		–
Dihombo	5.76	(4.48, 7.41)***		–
Luhindo	4.9	(3.8, 6.33)***		–
Dakawa	3.34	(2.56, 4.36)***		–
Mtibwa	1.00			
Water shaded area				
High	13.3	(10.59, 16.71)***	15.77	(6.66, 37.36)***
Medium–Low	4.66	(3.7, 5.88)***	4.81	(2.03, 11.4)***
Dry	1.00		1.00	
Ecosystem^a^				
Savannah	10.94	(8.73, 13.71)***	1.00	–
Rice-irrigation	4.12	(3.24, 5.24)***		–
Sugarcane	1.00		1.00	–

***p < 0.001, **p < 0.01

^a^Not included in the multiple regression model due to high correlation or multicollinearity

**Fig. 4:**
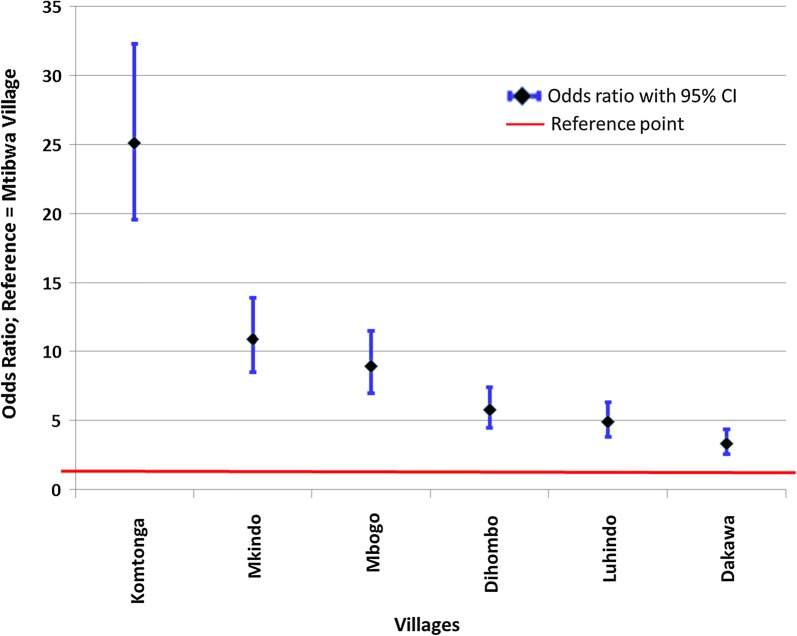
Trends of risk of malaria by villages arranged in descending order of water shaded area

Sleeping under a mosquito net reduced the risk of getting malaria by 26%. Season of the year was significantly associated with the rate of infection. Comparing between dry and hot seasons, risk of malaria was highest during dry and cool season (OR = 1.98, 95% CI 1.71, 2.3). Living in high water shaded areas increased the chances of getting malaria up to 15 times compared to living in dry shaded areas. Similarly, results obtained in the bivariate analysis also indicated that living in savannah and rice-irrigation areas were significantly increasing the risk of malaria as compared to living in sugarcane plantations. However, geometric mean parasite density was highest among residents of sugarcane ecosystem (434.25/μl) and lowest in the savannah (Dakawa = 161.44**/**μl). Trend of the risk by village indicates a high (25 times) and significant odds for malaria infection for individuals living in Komtonga (highly wet shaded) than in Mtibwa (dry shaded). This risk reduced sharply in villages with lesser water shaded area (Fig. 4). The variance of village-specific errors was estimated at 0.13 (95% CI 0.05–0.4) indicating moderate variance.

## Discussion

In this study, about one-third of individuals in the district were infected with malaria parasites, children aged 6–9 years being the most affected group. A recent cross-sectional study among schoolchildren (6–13 years) in a neighbouring Morogoro Urban district in Tanzania by Nzobo et al. [68] reported a relatively lower malaria prevalence of 5.4%, with those aged 6–9 years reported to be the most affected. Unlike in this study where males were at higher risk of malaria, in a study in Morogoro Urban, females were more affected with malaria than males [68]. In a hospital-based study in a western town of Kisumu in Kenya, malaria parasitaemia levels were significantly higher in males than females [69], indicating that males are probably more vulnerable to malaria infection than females. The difference between these findings is likely to be attributed to socio-behavioural factors which differ between rural and urban settings. In all villages, *P. falciparum* was the most important malaria species. Like many other parts of Tanzania, the prevalences of *P. malariae* or *P. ovale* in this area were low [70].

The prevalence of malaria varied strongly between age groups and the agro-ecosystems. Malaria prevalence was highest among communities in rice-irrigation ecosystem. Similar findings have been reported in Burundi [20], Mali [71, 72], Tanzania [22, 73], Cameroon [74] and Sri Lanka [49]. These studies reported that prevalence of malaria was highest in irrigated than in non-irrigated cultivation and this was also associated with large number of malaria mosquitoes. Results from this work indicated significant variation in level of parasitaemia even between different rice-irrigation practices. Malaria prevalence was higher among residents in traditional flooding rice irrigation (Komtonga) than improved rice-irrigation (Mkindo). An entomological survey conducted in the same area indicated variations in mosquito populations where a significant large number of anophelines mosquitoes (particularly *An. gambiae*) and entomological inoculation rate were observed in areas practicing flooded rice irrigation [34].

Irrigation rice fields are known to provide ideal breeding sites for *An. gambiae*, the main malaria vector in Africa [21], while *An. funestus* found commonly in savannah [37]. Irrigated-rice cultivation, depending of the number of cropping cycles, may also extend their breeding season and hence increase the annual duration for malaria transmission [22]. The variations in malaria prevalence within the study district were likely to be due to differences in ecological factors and socio-economic activities which affect malaria transmission risk. Various studies in Tanzania and elsewhere have shown that malaria transmission risk varies even between areas in close proximity [75–77]. Contrary to findings from this study, other studies in East Africa [31, 33] and West Africa [74, 78–80] have reported lower prevalence of malaria among rice-irrigated households than in non-irrigation villages, attributed to improved socio-economic status. Improved socioeconomic status due to rice growing in a study in northern Tanzania [73] was attributed to the reduced malaria prevalence, in spite of increased mosquito populations among villages adjacent to flooded rice fields. This could be explained by the widespread use of interventions by the rice farming community who used the income generated from agricultural production for malaria control, more specific for children [22]. Other factors could be differences in vector species and their feeding practices [32]. These findings emphasize the need for multi-sectoral collaborations while implementing development projects and malaria control strategies, for instance understanding transmission patterns, vector population and population behaviours [81].

In this study, communities living in sugarcane ecosystem had the lowest malaria parasitaemia. Only a few incidence of increase in malaria has been associated with sugarcane in Africa. It is known that a properly maintained sugarcane irrigation system does not offer appropriate breeding sites for malaria vectors [82]. Usually, sugarcane irrigation does not support large expanses of water surfaces. Moreover, under normal conditions, the thick vegetation cover created by sugarcane growth would limit the numbers of *An. gambiae* because of this species preference for open breeding sites [34].

This study included school-children tested for malaria while at school. As there were no restrictions in registration of students, it is possible, though less likely, that some children were included in estimation of malaria prevalence in village different to where they actually reside, which could influence prevalence levels observed.

This study demonstrated that about half of the children involved were anaemic. The findings show that moderate anaemia was common in children in all villages. Children in the sugarcane plantation had the lowest prevalence of anaemia than those in other agro-ecosystems. Like in Mvomero district, anaemia is a major health problem in Tanzania, especially among young children. However, a similar study in a neighbouring district of Morogoro Urban, reported a relatively lower anaemia prevalence of 10.1% [68]. The findings that the prevalence of anaemia among children in the savannah ecosystems was higher than in irrigation ecosystem (with higher malaria prevalence) suggest that anaemia in this area is most likely to be the result of dietary deficiency. Population based studies in Tanzania have indicated that the most common cause of anaemia among children in Tanzania is nutritional anaemia [83]. Communities in Dakawa and Luhindo are pastoralists and agro-pastoralists with a significant number working in large rice farms. This livelihood had been associated with unstable income and poor family care practices where either women are left home with less income while men moving with animals or children left on their own while both parents working out. Most families in the sugarcane plantation agro-ecosystem work in the factory where stable income and probably more time to provide childcare could be the case. The findings of the current study are similar to the national figures of anaemia in Tanzania where almost three quarters of the children were reported anaemic; 25% with mild anaemia, 43% with moderate anaemia, and 4% with severe anaemia in 2012 [83]. However the situation has significantly improved as reported by a recent survey of 2016 [9]. On the other hand, enlargement of spleen was high in a Savannah village with low malaria infection rate which might indicate delays in care seeking for malaria treatment [84, 85].

High water shed was associated with higher malaria prevalence in this study. High water sheds provide conditions such as vegetation cover, temperature, and humidity conditions that are conducive to distribution and survival of malaria vectors. The fact that Komtonga village is characterized by flooding water almost throughout the year, provide suitable mosquito breeding habitats [34] and hence community is at higher risk of malaria infection than those with fewer/less water shaded conditions.

## Conclusions

There are significant variations in malaria parasitaemia prevalence among individuals living in different agro-ecological zones within small geographical localities. Such variations are likely to be attributed to different transmission levels and socio-economic factors. The variability of the levels of parasitaemia illustrates the existing of different micro-ecological zones, hence the spatial and temporal variation in malaria transmission. It is therefore important, that malaria control/elimination strategies are designed to focus on specific local needs and those who practice irrigation agriculture need to be well informed of the consequences of their farming practices. National and sub-national malaria control programmes need to allocate resources for malaria control proportionate to risk of malaria transmission in specific localities.

## Data Availability

The datasets generated and used for this manuscript are available from the corresponding author on reasonable request.

## Abbreviations

AIC: Akaike information criteria
CI: confidence interval
GMPD: geometric mean parasite density
Hb: haemoglobin
ITN: insecticide-treated nets
IQR: interquartile range
NIMR: National Institute for Medical Research
OR: odds ratio
TDHS-MIS: Tanzania Demographic and Health Survey and Malaria Indicator Survey
VIF: variance inflation factor
WHO: World Health Organization

## References

[1] WHO (2017). World Malaria Report 2017.

[2] WHO (2016). World malaria report 2016.

[3] Mabey D, Peeling R, Ustianowski A (2004). Tropical infectious diseases: diagnostics for the developing world. Nat Rev Microbiol.

[4] Snow R, Guerra C, Noor A, Myint H, Hay S (2005). The global distribution of clinical episodes of *Plasmodium falciparum* malaria. Nature.

[5] Idro R, Aketch S, Gwer S, Newton C (2006). Research priorities in the management of severe *Plasmodium falciparum* malaria in children. Ann Trop Med Parasitol.

[6] Hagenlocher M, Castro MC, Gething P, Patil A, Smith D, Guerra C (2015). Mapping malaria risk and vulnerability in the United Republic of Tanzania: a spatial explicit model. Popul Health Metr.

[7] WHO (2011). Haemoglobin concentrations for the diagnosis of anaemia and assessment of severity. Vitamin and mineral nutrition information system.

[8] Winskill P, Rowland M, Mtove G, Malima RC, Kirby MJ (2011). Malaria risk factors in north-east Tanzania. Malar J.

[9] TDHS-MIS, Tanzania Demographic and Health Survey and Malaria Indicator Survey. Dar es Salaam, Tanzania and Rockville, Maryland, USA; 2016.

[10] Mboera LEG, Rumisha SF, Lyimo EP, Chiduo MG, Mangu CD, Mremi IR (2018). Cause-specific mortality patterns among hospital deaths in Tanzania, 2006–2015. PLoS ONE.

[11] Korenromp EL, Armstrong-Schellenberg JRM, Williams BG, Nahlen BL, Snow RW (2004). Impact of malaria control on childhood anaemia in Africa—a quantitative review. Trop Med Int Health..

[12] Ronald LA, Kenny SL, Klinkenberg E, Akoto AO, Boakye I, Barnish G (2006). Malaria and anaemia among children in two communities of Kumasi, Ghana: a cross-sectional survey. Malar J.

[13] Mazigo HD, Rumisha SF, Chiduo MG, Bwana VM, Mboera LEG (2017). Malaria among rice farming communities in Kilangali village, Kilosa district, Central Tanzania: prevalence, intensity and associated factors. Infect Dis Poverty.

[14] Craig MH, Snow RW, le Sueur D (1999). A climate-based distribution model of malaria transmission in sub-Saharan Africa. Parasitol Today.

[15] Patz JA, Graczyk TK, Geller N, Vittor AY (2000). Effects of environmental change on emerging parasitic diseases. Int J Parasitol.

[16] Minakawa N, Sonye G, Mogi M, Githeko A, Yan G (2002). The effects of climatic factors on the distribution and abundance of malaria vectors in Kenya. J Med Entomol.

[17] Adu-Prah S, Kofi Tetteh E (2015). Spatiotemporal analysis of climate variability impacts on malaria prevalence in Ghana. Appl Geogr.

[18] Chirebvu E, Chimbari MJ, Ngwenya BN, Sartorius B (2016). Clinical malaria transmission trends and its association with climatic variables in Tubu village, Botswana: a retrospective analysis. PLoS ONE.

[19] Hunter JM, Rey L, Scott D (1982). Man-made lakes and man-made diseases. Towards a policy resolution. Soc Sci Med.

[20] Coosemans M (1985). Comparison of malarial endemicity in a rice-growing zone and in a cotton-growing zone in the Rusizi Plain, Burundi. Ann Soc Belg Med Trop.

[21] Carnevale P, Guillet P, Robert V, Fontenille D (1999). Diversity of malaria in rice growing areas of the Afrotropical region. Parassitologia.

[22] Ijumba J, Lindsay S (2001). Impact of irrigation on malaria in Africa: paddies paradox. Med Vet Entomol.

[23] Worrall E, Basu S, Hanson K (2005). The relationship between socio-economic status and malaria: a review of the literature. Trop Med Int Health.

[24] Keiser J, De Castro MC, Maltese MF, Bos R, Tanner M, Singer BH (2005). Effect of irrigation and large dams on the burden of malaria on a global and regional scale. Am J Trop Med Hyg.

[25] Baragatti M, Fournet F, Henry M-C, Assi S, Ouedraogo H, Rogier C (2009). Social and environmental malaria risk factors in urban areas of Ouagadougou, Burkina Faso. Malar J.

[26] Deressa W, Ali A, Berhane Y (2006). Review of the interplay between population dynamics and malaria transmission in Ethiopia. Ethiop J Health Dev.

[27] Hawkes C, Ruel M (2006). The links between agriculture and health: an intersectoral opportunity to improve the health and livelihoods of the poor. Bull World Health Organ.

[28] Yasuoka J, Levins R (2007). Impact of deforestation and agricultural development on anopheline ecology and malaria epidemiology. Am J Trop Med Hyg.

[29] Randell H, Dickinson K, Shayo E, Mboera L (2010). Environmental management for malaria control: knowledge and practices in Mvomero, Tanzania. Ecohealth.

[30] Kebede A, McCann JC, Kiszewski AE, Ye-Ebiyo Y (2005). New evidence of the effects of agro-ecologic change on malaria transmission. Am J Trop Med Hyg.

[31] Mutero CM, McCartney M, Boelee E (2006). Agriculture, malaria and water-associated diseases: understanding the links between agriculture and health.

[32] Mutero CM, Kabutha C, Kimani V, Kabuage L, Gitau G, Ssennyonga J (2004). A transdisciplinary perspective on the links between malaria and agroecosystems in Kenya. Acta Trop.

[33] Ijumba JN, Mosha FW, Lindsay SW (2002). Malaria transmission risk variations derived from different agricultural practices in an irrigated area of northern Tanzania. Med Vet Entomol.

[34] Mboera LEG, Senkoro KP, Mayala BK, Rumisha SF, Rwegoshora RT, Mlozi MRS (2010). Spatio-temporal variation in malaria transmission intensity in five agro-ecosystems in Mvomero district, Tanzania. Geospat Health.

[35] Muturi EJ, Mwangangi J, Shililu J, Muriu S, Jacob B, Kabiru E (2007). Mosquito species succession and physicochemical factors affecting their abundance in rice fields in Mwea, Kenya. J Med Entomol.

[36] Diuk-Wasser MA, Touré MB, Dolo G, Bagayoko M, Sogoba N, Sissoko I (2007). Effect of rice cultivation patterns on malaria vector abundance in rice-growing villages in Mali. Am J Trop Med Hyg.

[37] Cohuet A, Simard F, Wondji CS, Antonio-Nkondjio C, Awono-Ambene P, Fontenille D (2004). High malaria transmission intensity due to *Anopheles funestus* (Diptera: Culicidae) in a village of savannah–forest transition area in Cameroon. J Med Entomol.

[38] Oomen JMV, de Wold J, Jobin WR (1988). Health and irrigation: incorporation of disease control measures in irrigation: a multi-faceted task in design, construction and operations.

[39] Norris DE (2004). Mosquito-borne diseases as a consequence of land use change. EcoHealth.

[40] Patz JA, Daszak P, Tabor GM, Aguirre AA, Pearl M, Epstein J (2004). Unhealthy landscapes: policy recommendations on land use change and infectious disease emergence. Environ Health Perspect.

[41] Stevenson JC, Stresman GH, Gitonga CW, Gillig J, Owaga C, Marube E (2013). Reliability of school surveys in estimating geographic variation in malaria transmission in the Western Kenyan highlands. PLoS ONE.

[42] World Bank Group. Tanzania Mainland Poverty Assessment. 2015.

[43] Shikuku KM, Winowiecki L, Twyman J, Eitzinger A, Perez JG, Mwongera C (2017). Smallholder farmers’ attitudes and determinants of adaptation to climate risks in East Africa. Clim Risk Manag.

[44] Menozzi D, Fioravanzi M, Donati M (2015). Farmer’s motivation to adopt sustainable agricultural practices. Bio-based Appl Econ.

[45] Kibret S, Alemu Y, Boelee E, Tekie H, Alemu D, Petros B (2009). The impact of a small-scale irrigation scheme on malaria transmission in Ziway area, Central Ethiopia. Trop Med Int Health.

[46] Wielgosz B, Kato E, Ringler C (2014). Agro-ecology, household economics and malaria in Uganda: empirical correlations between agricultural and health outcomes. Malar J.

[47] Jaleta K, Hill S, Seyoum E (2013). Agro-ecosystems impact malaria prevalence: large-scale irrigation drives vector population in western Ethiopia. Malar J.

[48] Mboera L, Mazigo HD, Rumisha S, Kramer R (2013). Towards malaria elimination and its implication for vector control, disease management and livelihoods in Tanzania. MalarWorld J.

[49] Klinkenberg E, van der Hoek W, Amerasinghe FP (2004). A malaria risk analysis in an irrigated area in Sri Lanka. Acta Trop.

[50] Klinkenberg E, McCall PJ, Hastings IM, Wilson MD, Amerasinghe FP, Donnelly MJ (2005). Malaria and irrigated crops, Accra, Ghana. Emerg Infect Dis.

[51] Mboera LEG, Senkoro KP, Rumisha SF, Mayala BK, Shayo EH, Mlozi MRS (2011). *Plasmodium falciparum* and helminth coinfections among schoolchildren in relation to agro-ecosystems in Mvomero district, Tanzania. Acta Trop.

[52] Asenso-Okyere K, Asante F, Tarekegn J, Andam K (2009). The linkages between agriculture and malaria: issues for policy, research, and capacity strengthening.

[53] Gebreslasie MT (2015). A review of spatial technologies with applications for malaria transmission modelling and control in Africa. Geospat Health.

[54] National Bureau of Statistics Ministry of Finance (2013). United Republic of Tanzania.

[55] Daniel W (1999). Biostatistics: a foundation for analysis in the health sciences.

[56] Lwanga S, Lemeshow S. Sample size determination in health studies: a practical manual. 1991.

[57] Naing L, Winn T, Rusli BN (2006). Practical issues in calculating the sample size for prevalence studies. Arch Orofac Sci.

[58] Lemnge MM, Msangeni HA, Rønn AM, Salum FM, Jakobsen PH, Mhina JI (1997). Maloprim malaria prophylaxis in children living in a holoendemic village in north-eastern Tanzania. Trans R Soc Trop Med Hyg.

[59] Kazadi W, Sexton JD, Bigonsa M, W’Okanga B, Way M (2004). Malaria in primary school children and infants in kinshasa, democratic republic of the congo: surveys from the 1980s and 2000. Am J Trop Med Hyg.

[60] Deloron P, Ringwald P, Luty AJ, Renaut A, Minh TN, Mbessy JR (1999). Relationships between malaria prevalence and malaria-related morbidity in school children from two villages in central Africa. Am J Trop Med Hyg.

[61] Ekpenyong EA, Eyo JE (2008). Malaria control and treatment strategies among school children in semi-urban tropical communities. West Indian Med J.

[62] Kapesa A, Kweka EJ, Zhou G, Atieli HE, Kamugisha E, Mazigo HD (2018). Utility of passive malaria surveillance in hospitals as a surrogate to community infection transmission dynamics in western Kenya. Arch Public Health.

[63] Mboera L, Shayo E, Senkoro K, Rumisha S, Mlozi MR, Mayala BK (2010). Knowledge, perceptions and practices of farming communities on linkages between malaria and agriculture in Mvomero district, Tanzania. Acta Trop.

[64] Gilles H, Warrell D (1993). Bruce-Chwatt’s essential malariology.

[65] Cuzick J (1985). A Wilcoxon-type test for trend. Stat Med.

[66] Akaike H (1992). Information theory and an extension of the maximum likelihood principle. Breakthrough in Statistics.

[67] Farrar D, Glauber R (1967). Multicollinearity in regression analysis: the problem revisited. Rev Econ Stat.

[68] Nzobo BJ, Ngasala BE, Kihamia CM (2015). Prevalence of asymptomatic malaria infection and use of different malaria control measures among primary school children in Morogoro Municipality, Tanzania. Malar J.

[69] Ng’ong’a G, Sharma R, Gicheru M, Odour M, Vulule J. Influence of age and sex on *Plasmodium falciparum* malaria susceptibility in children 1–10 year in Kisumu Town, Kenya. Kenyatta University Repository. 2011.

[70] Mboera LEG. Fifty years of health research in Tanzania (1949–1999). Annotated Bibliography. DUP (1996) Ltd; 2000.

[71] Klinkenberg E, Takken W, Huibers F, Touré YT (2003). The phenology of malaria mosquitoes in irrigated rice fields in Mali. Acta Trop.

[72] Sogoba N, Doumbia S, Vounatsou P, Bagayoko MM, Dolo G, Traoré SF (2007). Malaria transmission dynamics in Niono, Mali: the effect of the irrigation systems. Acta Trop.

[73] Ijumba JN, Shenton FC, Clarke SE, Mosha FW, Lindsay SW (2002). Irrigated crop production is associated with less malaria than traditional agricultural practices in Tanzania. Trans R Soc Trop Med Hyg.

[74] Audibert M, Josseran R, Josse R, Adjidji A (1990). Irrigation, schistosomiasis, and malaria in the Logone Valley, Cameroon. Am J Trop Med Hyg.

[75] Yé Y, Kyobutungi C, Louis VR, Sauerborn R (2007). Micro-epidemiology of *Plasmodium falciparum* malaria: is there any difference in transmission risk between neighbouring villages?. Malar J.

[76] Wang S-J, Lengeler C, Mtasiwa D, Mshana T, Manane L, Maro G (2006). Rapid Urban Malaria Appraisal (RUMA) II: epidemiology of urban malaria in Dar es Salaam (Tanzania). Malar J.

[77] Wang S-J, Lengeler C, Smith TA, Vounatsou P, Cissé G, Diallo DA (2005). Rapid urban malaria appraisal (RUMA) in sub-Saharan Africa. Malar J.

[78] Faye O, Fontenille D, Gaye O, Sy N, Molez JF, Konate L (1995). Malaria and rice growing in the Senegal River delta (Senegal). Ann Soc Belg Med Trop.

[79] Lindsay S, Wilkins H, Zieler H, Daly R (1991). Ability of *Anopheles gambiae* mosquitoes to transmit malaria during the dry and wet seasons in an area of irrigated rice cultivation in The Gambia. J Trop Med Hyg.

[80] Sissoko MS, Dicko A, Briët OJT, Sissoko M, Sagara I, Keita HD (2004). Malaria incidence in relation to rice cultivation in the irrigated Sahel of Mali. Acta Trop.

[81] Sanchez-Ribas J, Parra-Henao G, Guimarães AÉ (2012). Impact of dams and irrigation schemes in Anopheline (Diptera: Culicidae) bionomics and malaria epidemiology. Rev Inst Med Trop Sao Paulo.

[82] Packard R (1986). Agricultural development, migrant labor and the resurgence of malaria in Swaziland. Soc Sci Med.

[83] TDHS, Tanzania Demographic and Health Survey. Dar es Salaam, United Republic of Tanzania; 2005.

[84] Ozsoy MF, Oncul O, Pekkafali Z, Pahsa A, Yenen OS (2004). Splenic complications in malaria: report of two cases from Turkey. J Med Microbiol.

[85] Zingman BS, Viner BL (1993). Splenic complications in malaria: case report and review. Clin Infect Dis.

